# Correction: An invasive disease, sylvatic plague, increases fragmentation of black-tailed prairie dog (*Cynomys ludovicianus*) colonies

**DOI:** 10.1371/journal.pone.0261903

**Published:** 2021-12-21

**Authors:** The PLOS ONE Staff

## Notice of republication

This article was republished on December 10, 2020, to remove an erroneous ’Paperpile’ link that appeared in the Introduction paragraph of the online and PDF versions of the article. The publisher apologizes for the error. Please download this article again to view the correct version. The originally published, uncorrected article and the republished, corrected article are provided here for reference.

## Supporting information

S1 File Originally published, uncorrected article. (PDF)

S2 File Republished, corrected article. (PDF)
