# Supplementary material for: An invasive disease, sylvatic plague, increases fragmentation of black-tailed prairie dog (Cynomys ludovicianus) colonies
Source: PLoS One. 2020 Jul 23;15(7):e0235907. doi: 10.1371/journal.pone.0235907 (PMC7377483; doi:10.1371/journal.pone.0235907)
Supplement: S1 Table — (DOCX) [file pone.0235907.s001.docx]

**Any use of trade, firm, or product names is for descriptive purposes only and does not imply endorsement by the U.S. Government.S1 Table: Comparison between the fitted and intercept model for the selected spatial characteristics of each colony.**

| **Spatial characteristic** | **ΔAIC from random effects only model** | **Years since plague arrived coefficient estimate (95% CI)** |
| --- | --- | --- |
| log(CA) | 41.52 | -0.109 (-0.141, -0.077) |
| NP* | 221.98 | 0.111 (0.097, 0.125) |
| log(AREA_MN) | 88.07 | -0.179 (-0.215, -0.144) |
| SHAPE_MN | 3.92 | -0.012 (-0.022, -.002) |
| CONTIG_MN | 123.49 | -0.034 (-0.039, -0.028) |
| log(PROX_MN) | -1.982 | NA |

Log: natural log. NP: number of patches, CA: class area, AREA_MN: mean area of a patch within a colony complex, SHAPE_M: mean shape of patches within a colony complex, CONTIG_MN: mean contiguous index of patches in a colony complex, PROX_MN: mean proximity index of a patch within a colony complex or within the total landscape.

* This model was fit using a Poisson distribution.
