# Supplementary figures and images for: An invasive disease, sylvatic plague, increases fragmentation of black-tailed prairie dog (Cynomys ludovicianus) colonies
Source: PLoS One. 2020 Jul 23;15(7):e0235907. doi: 10.1371/journal.pone.0235907 (PMC7377483; doi:10.1371/journal.pone.0235907)

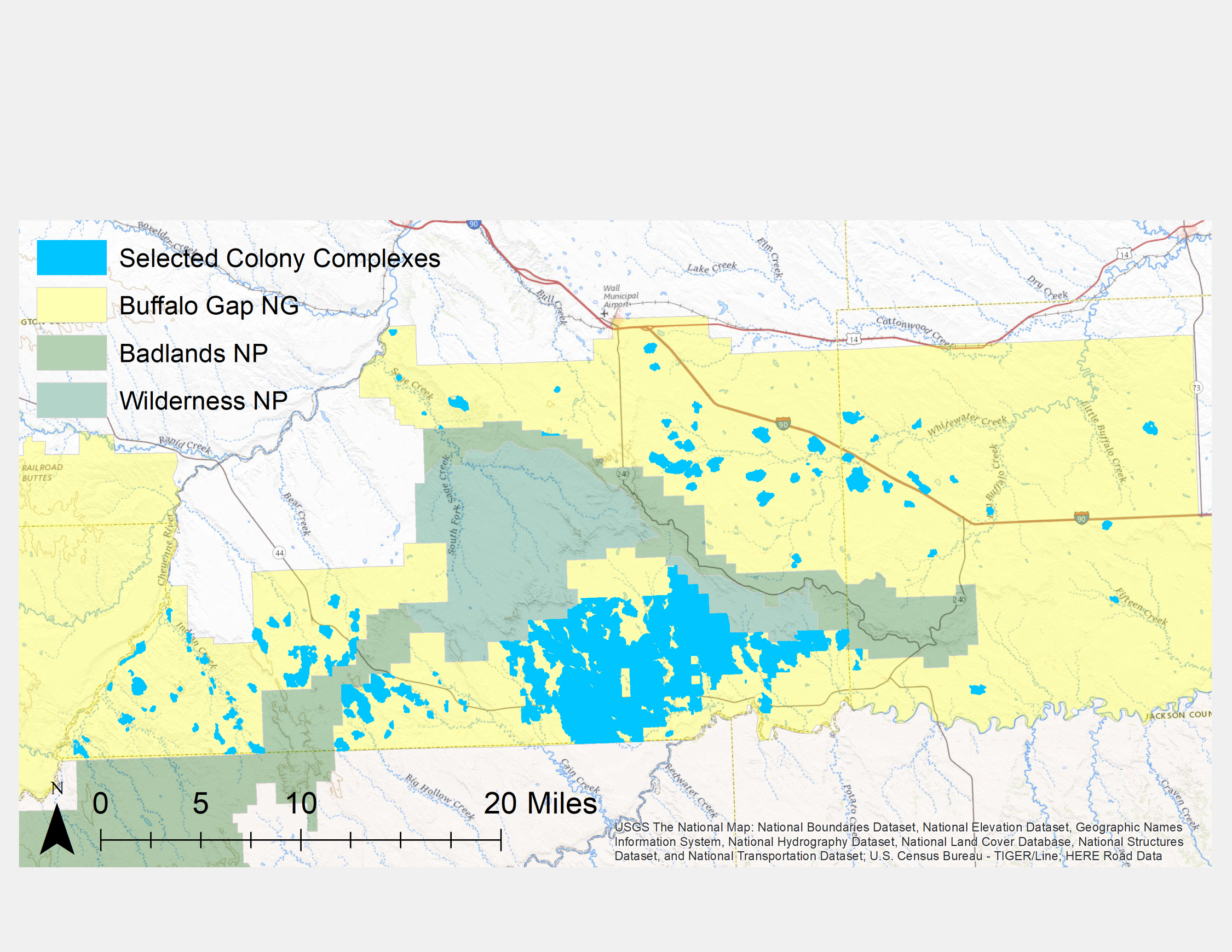

Supplement: S1 Fig — The blue shapes represent the buffered colony boundaries used for identifying colonies that belonged to the same complex through the years. Base map and public land boundaries are from the USGS National Map Server (https://viewer.nationalmap.gov/advanced-viewer/). (TIF) [file pone.0235907.s002.tif]

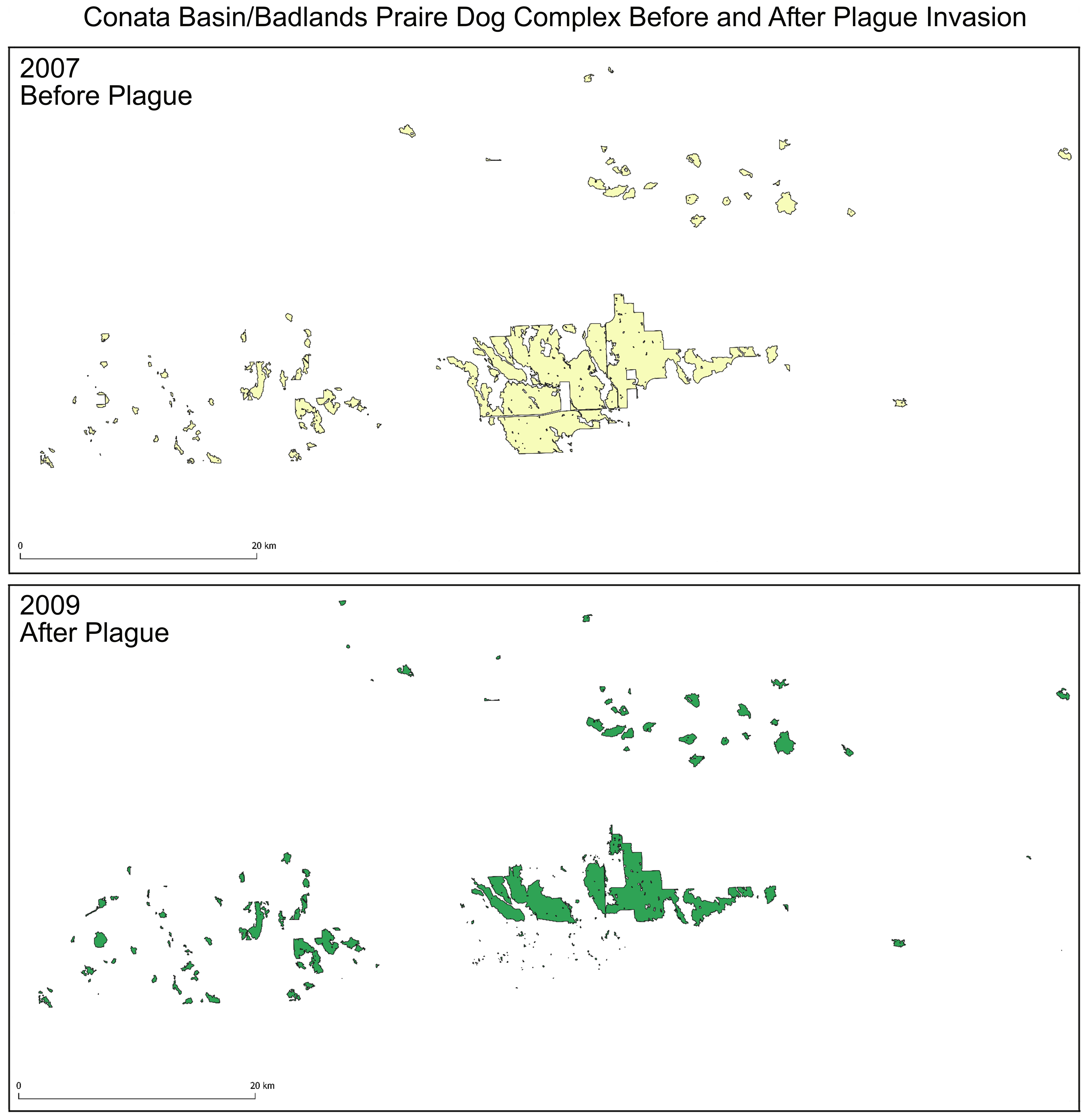

Supplement: S2 Fig — (TIF) [file pone.0235907.s003.tif]
