# Supplementary material for: An invasive disease, sylvatic plague, increases fragmentation of black-tailed prairie dog (Cynomys ludovicianus) colonies
Source: PLoS One. 2020 Jul 23;15(7):e0235907. doi: 10.1371/journal.pone.0235907 (PMC7377483; doi:10.1371/journal.pone.0235907)
Supplement: S2 File — In this file the generalized linear models are ran for the full data set and the reduced dataset (Colony 3 excluded, and aerial surveys [1993, 2006] excluded). (HTML) [file pone.0235907.s005.html]

Supplement Keuler et al. An invasive disease, sylvatic plague, increases fragmentation of black-tailed prairie dog (Cynomys ludovicianus) colonies.


# Supplement Keuler et al. An invasive disease, sylvatic plague, increases fragmentation of black-tailed prairie dog (Cynomys ludovicianus) colonies.

#### G.M. Bron and K.L.D. Richgels

#### 6/19/2020

Supplement to An invasive disease, sylvatic plague, increases fragmentation of black-tailed prairie dog (*Cynomys ludovicianus*) colonies.

In this file the generalized linear models are ran for the full data set and the reduced dataset (Colony 3 excluded, and aerial surveys [1993,2006] excluded).

DISCLAIMER: Any use of trade, firm, or product names is for descriptive purposes only and does not imply endorsement by the U.S. Government. This software has been approved for release by the U.S. Geological Survey (USGS). Although the software has been subjected to rigorous review, the USGS reserves the right to update the software as needed pursuant to further analysis and review. No warranty, expressed or implied, is made by the USGS or the U.S. Government as to the functionality of the software and related material nor shall the fact of release constitute any such warranty. Furthermore, the software is released on condition that neither the USGS nor the U.S. Government shall be held liable for any damages resulting from its authorized or unauthorized use.

# Data files

Three data files are used in this analysis:

- Landscape data for 12 years of data collection
- Colony level data \*\* Complete data set \*\* Data set with colonies that experienced plague only \*\* Subset of the data without colony 3 (the largest colony by far) \*\* Subset of the data without 1993 and 2006, as it is was surveiled by air

## Basic regression models

### Colony Area

Here the log transformed area of each colony is regressed against the number of years post plague, the colony ID is used as a random effect to account for repeated measures.

```
## [1] 41.52002
```

**Colony area of plague affected colonies only**

In the following part only the colonies that were affected by plague are included.

```
## Linear mixed model fit by maximum likelihood  ['lmerMod']
## Formula: log(CA) ~ yPI + (1 | colonyID)
##    Data: df.s
## 
##      AIC      BIC   logLik deviance df.resid 
##    557.2    569.9   -274.6    549.2      175 
## 
## Scaled residuals: 
##     Min      1Q  Median      3Q     Max 
## -3.2963 -0.4850  0.1434  0.6511  1.7213 
## 
## Random effects:
##  Groups   Name        Variance Std.Dev.
##  colonyID (Intercept) 2.7515   1.6588  
##  Residual             0.7968   0.8927  
## Number of obs: 179, groups:  colonyID, 26
## 
## Fixed effects:
##             Estimate Std. Error t value
## (Intercept)  3.32628    0.33828   9.833
## yPI         -0.22947    0.02659  -8.631
## 
## Correlation of Fixed Effects:
##     (Intr)
## yPI -0.177
```

```
## Computing profile confidence intervals ...
```

```
##                  2.5 %     97.5 %
## .sig01       1.2764244  2.2559996
## .sigma       0.8013688  1.0026593
## (Intercept)  2.6372945  4.0103636
## yPI         -0.2820192 -0.1770761
```

```
## [1] 59.36612
```

### Number of patches per colony

Here the number of patches of each colony per year is regressed against the number of years post plague, the colony ID is used as a random effect to account for repeated measures.

```
## Generalized linear mixed model fit by maximum likelihood (Laplace
##   Approximation) [glmerMod]
##  Family: poisson  ( log )
## Formula: NP ~ yPI + (1 | colonyID)
##    Data: df.c
## 
##      AIC      BIC   logLik deviance df.resid 
##   2165.6   2178.6  -1079.8   2159.6      564 
## 
## Scaled residuals: 
##     Min      1Q  Median      3Q     Max 
## -6.1318 -0.4483 -0.0986  0.2010  6.9698 
## 
## Random effects:
##  Groups   Name        Variance Std.Dev.
##  colonyID (Intercept) 0.5576   0.7467  
## Number of obs: 567, groups:  colonyID, 91
## 
## Fixed effects:
##             Estimate Std. Error z value Pr(>|z|)    
## (Intercept) 0.254540   0.089298    2.85  0.00437 ** 
## yPI         0.110821   0.007085   15.64  < 2e-16 ***
## ---
## Signif. codes:  0 '***' 0.001 '**' 0.01 '*' 0.05 '.' 0.1 ' ' 1
## 
## Correlation of Fixed Effects:
##     (Intr)
## yPI -0.237
```

```
## Computing profile confidence intervals ...
```

```
##                  2.5 %    97.5 %
## .sig01      0.64104145 0.8810246
## (Intercept) 0.07427724 0.4286416
## yPI         0.09679672 0.1247255
```

```
## [1] 221.9832
```

**Number of patches of plague affected colonies only**

In the following part only the colonies that were affected by plague are included.

```
## Generalized linear mixed model fit by maximum likelihood (Laplace
##   Approximation) [glmerMod]
##  Family: poisson  ( log )
## Formula: NP ~ yPI + (1 | colonyID)
##    Data: df.s
## 
##      AIC      BIC   logLik deviance df.resid 
##    956.9    966.5   -475.5    950.9      176 
## 
## Scaled residuals: 
##     Min      1Q  Median      3Q     Max 
## -6.0493 -0.5339 -0.1103  0.3041  6.5655 
## 
## Random effects:
##  Groups   Name        Variance Std.Dev.
##  colonyID (Intercept) 1.151    1.073   
## Number of obs: 179, groups:  colonyID, 26
## 
## Fixed effects:
##             Estimate Std. Error z value Pr(>|z|)    
## (Intercept) 0.642039   0.219499   2.925  0.00344 ** 
## yPI         0.118156   0.008053  14.673  < 2e-16 ***
## ---
## Signif. codes:  0 '***' 0.001 '**' 0.01 '*' 0.05 '.' 0.1 ' ' 1
## 
## Correlation of Fixed Effects:
##     (Intr)
## yPI -0.114
```

```
## Computing profile confidence intervals ...
```

```
##                 2.5 %    97.5 %
## .sig01      0.8272799 1.4576928
## (Intercept) 0.1903397 1.0815960
## yPI         0.1022423 0.1339012
```

```
## [1] 193.6963
```

### Mean patch size per colony

Here the mean size of the patches of each colony per year is regressed against the number of years post plague, the colony ID is used as a random effect to account for repeated measures.

```
## Linear mixed model fit by maximum likelihood  ['lmerMod']
## Formula: log(AREA_MN) ~ yPI + (1 | colonyID)
##    Data: df.c
## 
##      AIC      BIC   logLik deviance df.resid 
##   1863.2   1880.5   -927.6   1855.2      563 
## 
## Scaled residuals: 
##     Min      1Q  Median      3Q     Max 
## -3.6603 -0.4950  0.0688  0.5947  2.4969 
## 
## Random effects:
##  Groups   Name        Variance Std.Dev.
##  colonyID (Intercept) 1.350    1.162   
##  Residual             1.106    1.052   
## Number of obs: 567, groups:  colonyID, 91
## 
## Fixed effects:
##             Estimate Std. Error t value
## (Intercept)  2.25508    0.13712  16.447
## yPI         -0.17937    0.01811  -9.905
## 
## Correlation of Fixed Effects:
##     (Intr)
## yPI -0.297
```

```
## Computing profile confidence intervals ...
```

```
##                  2.5 %     97.5 %
## .sig01       0.9852185  1.3818970
## .sigma       0.9883116  1.1223314
## (Intercept)  1.9821230  2.5250131
## yPI         -0.2149913 -0.1438077
```

```
## [1] 0.8357966
```

```
## Computing profile confidence intervals ...
```

```
##                 2.5 %     97.5 %
## .sig01      2.6783971  3.9824493
## .sigma      2.6866944  3.0720080
## (Intercept) 7.2581356 12.4910594
## yPI         0.8065484  0.8660543
```

```
## [1] 88.07216
```

**Mean patch area of affected colonies only**

In the following part only the colonies that were affected by plague are included.

```
## Linear mixed model fit by maximum likelihood  ['lmerMod']
## Formula: log(AREA_MN) ~ yPI + (1 | colonyID)
##    Data: df.s
## 
##      AIC      BIC   logLik deviance df.resid 
##    580.7    593.5   -286.4    572.7      175 
## 
## Scaled residuals: 
##     Min      1Q  Median      3Q     Max 
## -3.0432 -0.5356  0.1526  0.5844  2.1004 
## 
## Random effects:
##  Groups   Name        Variance Std.Dev.
##  colonyID (Intercept) 1.131    1.063   
##  Residual             1.063    1.031   
## Number of obs: 179, groups:  colonyID, 26
## 
## Fixed effects:
##             Estimate Std. Error t value
## (Intercept)  2.72745    0.23421   11.64
## yPI         -0.32453    0.03063  -10.60
## 
## Correlation of Fixed Effects:
##     (Intr)
## yPI -0.294
```

```
## Computing profile confidence intervals ...
```

```
##                  2.5 %     97.5 %
## .sig01       0.7812314  1.4870708
## .sigma       0.9253846  1.1587644
## (Intercept)  2.2514017  3.2000267
## yPI         -0.3850124 -0.2641591
```

```
## [1] 83.09882
```

### Mean shape index per colony

Here the mean shape index ( 0.25\*perimeter / area^2) of each colony per year is regressed against the number of years post plague, the colony ID is used as a random effect to account for repeated measures.

```
## Linear mixed model fit by maximum likelihood  ['lmerMod']
## Formula: SHAPE_MN ~ yPI + (1 | colonyID)
##    Data: df.c
## 
##      AIC      BIC   logLik deviance df.resid 
##    379.1    396.5   -185.6    371.1      563 
## 
## Scaled residuals: 
##     Min      1Q  Median      3Q     Max 
## -3.2708 -0.6226 -0.0889  0.4349  4.7736 
## 
## Random effects:
##  Groups   Name        Variance Std.Dev.
##  colonyID (Intercept) 0.05459  0.2336  
##  Residual             0.08831  0.2972  
## Number of obs: 567, groups:  colonyID, 91
## 
## Fixed effects:
##              Estimate Std. Error t value
## (Intercept)  1.624730   0.030176   53.84
## yPI         -0.012411   0.005086   -2.44
## 
## Correlation of Fixed Effects:
##     (Intr)
## yPI -0.377
```

```
## Computing profile confidence intervals ...
```

```
##                  2.5 %       97.5 %
## .sig01       0.1942609  0.281881474
## .sigma       0.2793266  0.317003419
## (Intercept)  1.5646378  1.684049979
## yPI         -0.0224242 -0.002420355
```

```
## [1] 3.919106
```

**Mean shape size of plague affected colonies only**

In the following part only the colonies that were affected by plague are included.

```
## Linear mixed model fit by maximum likelihood  ['lmerMod']
## Formula: SHAPE_MN ~ yPI + (1 | colonyID)
##    Data: df.s
## 
##      AIC      BIC   logLik deviance df.resid 
##     74.2     86.9    -33.1     66.2      175 
## 
## Scaled residuals: 
##      Min       1Q   Median       3Q      Max 
## -2.19037 -0.66814 -0.02876  0.49123  3.07055 
## 
## Random effects:
##  Groups   Name        Variance Std.Dev.
##  colonyID (Intercept) 0.03366  0.1835  
##  Residual             0.06880  0.2623  
## Number of obs: 179, groups:  colonyID, 26
## 
## Fixed effects:
##              Estimate Std. Error t value
## (Intercept)  1.623743   0.044935  36.136
## yPI         -0.018681   0.007763  -2.406
## 
## Correlation of Fixed Effects:
##     (Intr)
## yPI -0.386
```

```
## Computing profile confidence intervals ...
```

```
##                   2.5 %       97.5 %
## .sig01       0.12394986  0.267115183
## .sigma       0.23533476  0.294851293
## (Intercept)  1.53288308  1.714358153
## yPI         -0.03399309 -0.003373372
```

```
## [1] 3.687826
```

### Mean contiguity index per colony

Here the mean contiguity index (0 is a 1 pixel patch, 1 means a contigous patch shape) of each colony per year is regressed against the number of years post plague, the colony ID is used as a random effect to account for repeated measures.

48 contiguous states would be close by 1, the complete United States would have a lower CONTIG index.

```
## Linear mixed model fit by maximum likelihood  ['lmerMod']
## Formula: CONTIG_MN ~ yPI + (1 | colonyID)
##    Data: df.c
## 
##      AIC      BIC   logLik deviance df.resid 
##   -312.5   -295.1    160.2   -320.5      563 
## 
## Scaled residuals: 
##     Min      1Q  Median      3Q     Max 
## -3.6255 -0.4233  0.1723  0.6108  2.2093 
## 
## Random effects:
##  Groups   Name        Variance Std.Dev.
##  colonyID (Intercept) 0.01028  0.1014  
##  Residual             0.02766  0.1663  
## Number of obs: 567, groups:  colonyID, 91
## 
## Fixed effects:
##              Estimate Std. Error t value
## (Intercept)  0.779377   0.014436   53.99
## yPI         -0.033747   0.002829  -11.93
## 
## Correlation of Fixed Effects:
##     (Intr)
## yPI -0.437
```

```
## Computing profile confidence intervals ...
```

```
##                   2.5 %      97.5 %
## .sig01       0.08036283  0.12607857
## .sigma       0.15625948  0.17749670
## (Intercept)  0.75072216  0.80779063
## yPI         -0.03930253 -0.02819065
```

```
## [1] 123.4891
```

**Mean contig index of plague affected colonies only**

In the following part only the colonies that were affected by plague are included.

```
## Linear mixed model fit by maximum likelihood  ['lmerMod']
## Formula: CONTIG_MN ~ yPI + (1 | colonyID)
##    Data: df.s
## 
##      AIC      BIC   logLik deviance df.resid 
##   -121.8   -109.1     64.9   -129.8      175 
## 
## Scaled residuals: 
##     Min      1Q  Median      3Q     Max 
## -2.5002 -0.4860  0.1257  0.6462  2.0407 
## 
## Random effects:
##  Groups   Name        Variance Std.Dev.
##  colonyID (Intercept) 0.01038  0.1019  
##  Residual             0.02325  0.1525  
## Number of obs: 179, groups:  colonyID, 26
## 
## Fixed effects:
##             Estimate Std. Error t value
## (Intercept)  0.78918    0.02537   31.11
## yPI         -0.04769    0.00451  -10.57
## 
## Correlation of Fixed Effects:
##     (Intr)
## yPI -0.397
```

```
## Computing profile confidence intervals ...
```

```
##                   2.5 %      97.5 %
## .sig01       0.06853705  0.14865268
## .sigma       0.13683406  0.17133566
## (Intercept)  0.73843068  0.84083253
## yPI         -0.05657400 -0.03876282
```

```
## [1] 80.9296
```

### Mean proximity index per colony

Here the mean proximity index (0 or larger, 0 = no neigbors) of each colony per year is regressed against the number of years post plague, the colony ID is used as a random effect to account for repeated measures.

If patches had no neighbors (i.e. if PROX\_MN = 0) they were removed from the analysis.

```
## Linear mixed model fit by maximum likelihood  ['lmerMod']
## Formula: log(PROX_MN) ~ yPI + (1 | colonyID)
##    Data: df.c.ss
## 
##      AIC      BIC   logLik deviance df.resid 
##   1011.1   1025.1   -501.5   1003.1      239 
## 
## Scaled residuals: 
##     Min      1Q  Median      3Q     Max 
## -2.7117 -0.5032  0.1159  0.6029  2.2898 
## 
## Random effects:
##  Groups   Name        Variance Std.Dev.
##  colonyID (Intercept) 2.336    1.529   
##  Residual             2.572    1.604   
## Number of obs: 243, groups:  colonyID, 69
## 
## Fixed effects:
##              Estimate Std. Error t value
## (Intercept)  1.861109   0.263958   7.051
## yPI         -0.005653   0.042915  -0.132
## 
## Correlation of Fixed Effects:
##     (Intr)
## yPI -0.505
```

```
## Computing profile confidence intervals ...
```

```
##                  2.5 %     97.5 %
## .sig01       1.1890170 1.95469525
## .sigma       1.4510226 1.78368175
## (Intercept)  1.3210217 2.38206713
## yPI         -0.0909757 0.07912334
```

```
## [1] -1.982875
```

**Mean proximity index of plague affected colonies only**

In the following part only the colonies that were affected by plague are included.

```
## Linear mixed model fit by maximum likelihood  ['lmerMod']
## Formula: log(PROX_MN) ~ yPI + (1 | colonyID)
##    Data: df.s.ss
## 
##      AIC      BIC   logLik deviance df.resid 
##    453.2    464.0   -222.6    445.2      104 
## 
## Scaled residuals: 
##     Min      1Q  Median      3Q     Max 
## -2.7291 -0.5532  0.1349  0.5473  1.9801 
## 
## Random effects:
##  Groups   Name        Variance Std.Dev.
##  colonyID (Intercept) 1.983    1.408   
##  Residual             2.756    1.660   
## Number of obs: 108, groups:  colonyID, 23
## 
## Fixed effects:
##             Estimate Std. Error t value
## (Intercept)  2.23584    0.41043   5.448
## yPI         -0.15390    0.06775  -2.272
## 
## Correlation of Fixed Effects:
##     (Intr)
## yPI -0.483
```

```
## Computing profile confidence intervals ...
```

```
##                  2.5 %      97.5 %
## .sig01       0.9643680  2.09088643
## .sigma       1.4440470  1.93434134
## (Intercept)  1.3781674  3.04807328
## yPI         -0.2891136 -0.01947294
```

```
## [1] 3.010791
```

This concludes the core analysis in the paper.

## Reduced dataset: Colony 3 removed

Below you find the same analysis, but now Colony 3 is removed it was significantly larger than the other colonies.

```
## Linear mixed model fit by maximum likelihood  ['lmerMod']
## Formula: log(CA) ~ yPI + (1 | colonyID)
##    Data: df.c90
## 
##      AIC      BIC   logLik deviance df.resid 
##   1752.8   1770.1   -872.4   1744.8      551 
## 
## Scaled residuals: 
##     Min      1Q  Median      3Q     Max 
## -4.7450 -0.3937  0.0887  0.5744  3.0982 
## 
## Random effects:
##  Groups   Name        Variance Std.Dev.
##  colonyID (Intercept) 1.8555   1.3622  
##  Residual             0.9002   0.9488  
## Number of obs: 555, groups:  colonyID, 90
## 
## Fixed effects:
##             Estimate Std. Error t value
## (Intercept)  2.45618    0.15474  15.873
## yPI         -0.10789    0.01654  -6.522
## 
## Correlation of Fixed Effects:
##     (Intr)
## yPI -0.242
```

```
## Computing profile confidence intervals ...
```

```
##                  2.5 %      97.5 %
## .sig01       1.1670724  1.60778451
## .sigma       0.8909525  1.01320454
## (Intercept)  2.1484095  2.76118404
## yPI         -0.1404504 -0.07540634
```

```
## [1] 38.94216
```

```
## Generalized linear mixed model fit by maximum likelihood (Laplace
##   Approximation) [glmerMod]
##  Family: poisson  ( log )
## Formula: NP ~ yPI + (1 | colonyID)
##    Data: df.c90
## 
##      AIC      BIC   logLik deviance df.resid 
##   1900.9   1913.9   -947.4   1894.9      552 
## 
## Scaled residuals: 
##     Min      1Q  Median      3Q     Max 
## -3.1446 -0.4242 -0.1071  0.1371  7.2861 
## 
## Random effects:
##  Groups   Name        Variance Std.Dev.
##  colonyID (Intercept) 0.2993   0.5471  
## Number of obs: 555, groups:  colonyID, 90
## 
## Fixed effects:
##             Estimate Std. Error z value Pr(>|z|)    
## (Intercept)  0.24581    0.07534   3.263   0.0011 ** 
## yPI          0.10604    0.01027  10.322   <2e-16 ***
## ---
## Signif. codes:  0 '***' 0.001 '**' 0.01 '*' 0.05 '.' 0.1 ' ' 1
## 
## Correlation of Fixed Effects:
##     (Intr)
## yPI -0.408
```

```
## Computing profile confidence intervals ...
```

```
##                  2.5 %    97.5 %
## .sig01      0.45964093 0.6572872
## (Intercept) 0.09266686 0.3914600
## yPI         0.08561982 0.1262685
```

```
## [1] 96.97278
```

```
## Linear mixed model fit by maximum likelihood  ['lmerMod']
## Formula: log(AREA_MN) ~ yPI + (1 | colonyID)
##    Data: df.c90
## 
##      AIC      BIC   logLik deviance df.resid 
##   1829.1   1846.4   -910.5   1821.1      551 
## 
## Scaled residuals: 
##     Min      1Q  Median      3Q     Max 
## -3.6361 -0.4939  0.0711  0.5927  2.4911 
## 
## Random effects:
##  Groups   Name        Variance Std.Dev.
##  colonyID (Intercept) 1.329    1.153   
##  Residual             1.118    1.057   
## Number of obs: 555, groups:  colonyID, 90
## 
## Fixed effects:
##             Estimate Std. Error t value
## (Intercept)  2.22792    0.13733  16.223
## yPI         -0.17635    0.01838  -9.597
## 
## Correlation of Fixed Effects:
##     (Intr)
## yPI -0.302
```

```
## Computing profile confidence intervals ...
```

```
##                  2.5 %     97.5 %
## .sig01       0.9755724  1.3733126
## .sigma       0.9929560  1.1293047
## (Intercept)  1.9545573  2.4983173
## yPI         -0.2125011 -0.1402657
```

```
## [1] 82.81049
```

```
## Linear mixed model fit by maximum likelihood  ['lmerMod']
## Formula: SHAPE_MN ~ yPI + (1 | colonyID)
##    Data: df.c90
## 
##      AIC      BIC   logLik deviance df.resid 
##    380.3    397.6   -186.2    372.3      551 
## 
## Scaled residuals: 
##     Min      1Q  Median      3Q     Max 
## -3.2526 -0.6197 -0.1006  0.4475  4.7326 
## 
## Random effects:
##  Groups   Name        Variance Std.Dev.
##  colonyID (Intercept) 0.05526  0.2351  
##  Residual             0.08967  0.2995  
## Number of obs: 555, groups:  colonyID, 90
## 
## Fixed effects:
##              Estimate Std. Error t value
## (Intercept)  1.623036   0.030606  53.030
## yPI         -0.011976   0.005173  -2.315
## 
## Correlation of Fixed Effects:
##     (Intr)
## yPI -0.380
```

```
## Computing profile confidence intervals ...
```

```
##                   2.5 %       97.5 %
## .sig01       0.19522821  0.283933961
## .sigma       0.28126837  0.319667131
## (Intercept)  1.56208720  1.683202810
## yPI         -0.02216206 -0.001813584
```

```
## [1] 3.328936
```

```
## Linear mixed model fit by maximum likelihood  ['lmerMod']
## Formula: CONTIG_MN ~ yPI + (1 | colonyID)
##    Data: df.c90
## 
##      AIC      BIC   logLik deviance df.resid 
##   -300.7   -283.5    154.4   -308.7      551 
## 
## Scaled residuals: 
##     Min      1Q  Median      3Q     Max 
## -3.6155 -0.4192  0.1682  0.6114  2.1902 
## 
## Random effects:
##  Groups   Name        Variance Std.Dev.
##  colonyID (Intercept) 0.01008  0.1004  
##  Residual             0.02797  0.1673  
## Number of obs: 555, groups:  colonyID, 90
## 
## Fixed effects:
##              Estimate Std. Error t value
## (Intercept)  0.781205   0.014514   53.82
## yPI         -0.033683   0.002871  -11.73
## 
## Correlation of Fixed Effects:
##     (Intr)
## yPI -0.443
```

```
## Computing profile confidence intervals ...
```

```
##                   2.5 %     97.5 %
## .sig01       0.07905317  0.1253685
## .sigma       0.15702438  0.1786496
## (Intercept)  0.75235864  0.8097581
## yPI         -0.03932253 -0.0280459
```

```
## [1] 119.6961
```

```
## Linear mixed model fit by maximum likelihood  ['lmerMod']
## Formula: log(PROX_MN) ~ yPI + (1 | colonyID)
##    Data: df.c90.ss
## 
##      AIC      BIC   logLik deviance df.resid 
##    959.7    973.5   -475.9    951.7      227 
## 
## Scaled residuals: 
##      Min       1Q   Median       3Q      Max 
## -2.63458 -0.50124  0.09592  0.64664  2.23963 
## 
## Random effects:
##  Groups   Name        Variance Std.Dev.
##  colonyID (Intercept) 1.871    1.368   
##  Residual             2.653    1.629   
## Number of obs: 231, groups:  colonyID, 68
## 
## Fixed effects:
##             Estimate Std. Error t value
## (Intercept) 1.769272   0.254598   6.949
## yPI         0.002806   0.044119   0.064
## 
## Correlation of Fixed Effects:
##     (Intr)
## yPI -0.537
```

```
## Computing profile confidence intervals ...
```

```
##                   2.5 %     97.5 %
## .sig01       1.02041735 1.79369861
## .sigma       1.46847708 1.81900809
## (Intercept)  1.24636238 2.27226021
## yPI         -0.08488351 0.08997295
```

```
## [1] -1.996007
```

```
## [1] -1.996007
```

## Reduced dataset: 2006 & 1993 removed

Below you find the same analysis, but now all observations from 2006 and 1993 are removed because the data collection method was different.

```
## [1] 67
```

```
## Linear mixed model fit by maximum likelihood  ['lmerMod']
## Formula: log(CA) ~ yPI + (1 | colonyID)
##    Data: df.c.no06
## 
##      AIC      BIC   logLik deviance df.resid 
##   1252.3   1268.3   -622.1   1244.3      398 
## 
## Scaled residuals: 
##     Min      1Q  Median      3Q     Max 
## -4.8656 -0.3908  0.1108  0.5729  1.8391 
## 
## Random effects:
##  Groups   Name        Variance Std.Dev.
##  colonyID (Intercept) 2.3132   1.5209  
##  Residual             0.8056   0.8976  
## Number of obs: 402, groups:  colonyID, 67
## 
## Fixed effects:
##             Estimate Std. Error t value
## (Intercept)  2.78904    0.19732  14.135
## yPI         -0.08946    0.01798  -4.975
## 
## Correlation of Fixed Effects:
##     (Intr)
## yPI -0.234
```

```
## Computing profile confidence intervals ...
```

```
##                  2.5 %      97.5 %
## .sig01       1.2804897  1.83663961
## .sigma       0.8337165  0.97000303
## (Intercept)  2.3962739  3.17985944
## yPI         -0.1248361 -0.05412706
```

```
## [1] 21.95105
```

```
## Generalized linear mixed model fit by maximum likelihood (Laplace
##   Approximation) [glmerMod]
##  Family: poisson  ( log )
## Formula: NP ~ yPI + (1 | colonyID)
##    Data: df.c.no06
## 
##      AIC      BIC   logLik deviance df.resid 
##   1637.6   1649.6   -815.8   1631.6      399 
## 
## Scaled residuals: 
##     Min      1Q  Median      3Q     Max 
## -5.9818 -0.4591 -0.0770  0.2345  6.5373 
## 
## Random effects:
##  Groups   Name        Variance Std.Dev.
##  colonyID (Intercept) 0.6689   0.8179  
## Number of obs: 402, groups:  colonyID, 67
## 
## Fixed effects:
##             Estimate Std. Error z value Pr(>|z|)    
## (Intercept) 0.338052   0.111002   3.045  0.00232 ** 
## yPI         0.115722   0.007629  15.168  < 2e-16 ***
## ---
## Signif. codes:  0 '***' 0.001 '**' 0.01 '*' 0.05 '.' 0.1 ' ' 1
## 
## Correlation of Fixed Effects:
##     (Intr)
## yPI -0.231
```

```
## Computing profile confidence intervals ...
```

```
##                 2.5 %    97.5 %
## .sig01      0.6872913 0.9903497
## (Intercept) 0.1137175 0.5552677
## yPI         0.1006371 0.1307040
```

```
## [1] 211.7045
```

```
## Linear mixed model fit by maximum likelihood  ['lmerMod']
## Formula: log(AREA_MN) ~ yPI + (1 | colonyID)
##    Data: df.c.no06
## 
##      AIC      BIC   logLik deviance df.resid 
##   1311.3   1327.3   -651.6   1303.3      398 
## 
## Scaled residuals: 
##     Min      1Q  Median      3Q     Max 
## -3.6838 -0.4946  0.1010  0.5656  2.4296 
## 
## Random effects:
##  Groups   Name        Variance Std.Dev.
##  colonyID (Intercept) 1.421    1.192   
##  Residual             1.045    1.022   
## Number of obs: 402, groups:  colonyID, 67
## 
## Fixed effects:
##             Estimate Std. Error t value
## (Intercept)  2.42265    0.16393   14.78
## yPI         -0.16337    0.02042   -8.00
## 
## Correlation of Fixed Effects:
##     (Intr)
## yPI -0.319
```

```
## Computing profile confidence intervals ...
```

```
##                  2.5 %     97.5 %
## .sig01       0.9864316  1.4574636
## .sigma       0.9494820  1.1050610
## (Intercept)  2.0966420  2.7470488
## yPI         -0.2035164 -0.1232426
```

```
## [1] 56.75088
```

```
## Linear mixed model fit by maximum likelihood  ['lmerMod']
## Formula: SHAPE_MN ~ yPI + (1 | colonyID)
##    Data: df.c.no06
## 
##      AIC      BIC   logLik deviance df.resid 
##    292.8    308.8   -142.4    284.8      398 
## 
## Scaled residuals: 
##     Min      1Q  Median      3Q     Max 
## -3.2456 -0.6171 -0.0797  0.4459  4.4939 
## 
## Random effects:
##  Groups   Name        Variance Std.Dev.
##  colonyID (Intercept) 0.06536  0.2557  
##  Residual             0.09067  0.3011  
## Number of obs: 402, groups:  colonyID, 67
## 
## Fixed effects:
##              Estimate Std. Error t value
## (Intercept)  1.674908   0.038216  43.827
## yPI         -0.012889   0.005988  -2.152
## 
## Correlation of Fixed Effects:
##     (Intr)
## yPI -0.400
```

```
## Computing profile confidence intervals ...
```

```
##                   2.5 %       97.5 %
## .sig01       0.20715581  0.317223430
## .sigma       0.27970415  0.325377265
## (Intercept)  1.59909974  1.750480857
## yPI         -0.02465742 -0.001119527
```

```
## [1] 2.601998
```

```
## Linear mixed model fit by maximum likelihood  ['lmerMod']
## Formula: CONTIG_MN ~ yPI + (1 | colonyID)
##    Data: df.c.no06
## 
##      AIC      BIC   logLik deviance df.resid 
##   -232.5   -216.5    120.2   -240.5      398 
## 
## Scaled residuals: 
##     Min      1Q  Median      3Q     Max 
## -3.0849 -0.4622  0.1772  0.6266  2.3502 
## 
## Random effects:
##  Groups   Name        Variance Std.Dev.
##  colonyID (Intercept) 0.01068  0.1033  
##  Residual             0.02637  0.1624  
## Number of obs: 402, groups:  colonyID, 67
## 
## Fixed effects:
##              Estimate Std. Error t value
## (Intercept)  0.781997   0.017256  45.318
## yPI         -0.031231   0.003211  -9.727
## 
## Correlation of Fixed Effects:
##     (Intr)
## yPI -0.472
```

```
## Computing profile confidence intervals ...
```

```
##                   2.5 %      97.5 %
## .sig01       0.07981520  0.13194945
## .sigma       0.15081699  0.17548284
## (Intercept)  0.74794160  0.81624127
## yPI         -0.03754082 -0.02490539
```

```
## [1] 80.89209
```

```
## Linear mixed model fit by maximum likelihood  ['lmerMod']
## Formula: log(PROX_MN) ~ yPI + (1 | colonyID)
##    Data: df.c.no06.ss
## 
##      AIC      BIC   logLik deviance df.resid 
##    799.6    812.7   -395.8    791.6      190 
## 
## Scaled residuals: 
##     Min      1Q  Median      3Q     Max 
## -2.7403 -0.5060  0.0421  0.5893  1.9957 
## 
## Random effects:
##  Groups   Name        Variance Std.Dev.
##  colonyID (Intercept) 1.740    1.319   
##  Residual             2.563    1.601   
## Number of obs: 194, groups:  colonyID, 54
## 
## Fixed effects:
##              Estimate Std. Error t value
## (Intercept)  2.166829   0.272871   7.941
## yPI         -0.009449   0.046161  -0.205
## 
## Correlation of Fixed Effects:
##     (Intr)
## yPI -0.544
```

```
## Computing profile confidence intervals ...
```

```
##                  2.5 %     97.5 %
## .sig01       0.9794323 1.75446766
## .sigma       1.4339835 1.80174767
## (Intercept)  1.6100328 2.70485811
## yPI         -0.1010991 0.08165224
```

```
## [1] -1.958452
```

```
## [1] -1.996007
```
