# Supplementary material for: An invasive disease, sylvatic plague, increases fragmentation of black-tailed prairie dog (Cynomys ludovicianus) colonies
Source: PLoS One. 2020 Jul 23;15(7):e0235907. doi: 10.1371/journal.pone.0235907 (PMC7377483; doi:10.1371/journal.pone.0235907)
Supplement: Attachment — Submitted filename: Rebuttal Letter.docx [file pone.0235907.s006.docx]

Dear Dr. Becker, Dr. Livieri and anonymous reviewer,

Thank you very much for your thorough review of our manuscript and detailed suggestions for its improvement.

We have tried to address all comments and provided a response below.

Best Regards,

Dr. Katherine Richgels

Also on behalf of co-authors

Journal Requirements:

1. When submitting your revision, we need you to address these additional requirements.

Please ensure that your manuscript meets PLOS ONE's style requirements, including those for file naming. The PLOS ONE style templates can be found at

<http://www.journals.plos.org/plosone/s/file?id=wjVg/PLOSOne_formatting_sample_main_body.pdf> and <http://www.journals.plos.org/plosone/s/file?id=ba62/PLOSOne_formatting_sample_title_authors_affiliations.pdf>

***Response:*** *Done.*

2. In your Methods section, please provide additional location information of the study area, including geographic coordinates for the data set if available.

***Response:*** *We have included additional description of the area covered in the methods and will include a shapefile of the colony boundaries selected for our analysis through time with the USGS data release. In addition, a map of the colony area was added as supplemental figure S1.*

3. In your Methods section, please provide additional information regarding the permits you obtained for the work. Please ensure you have included the full name of the authority that approved the field site access and, if no permits were required, a brief statement explaining why

***Response:***  *We included a statement explaining why permits were not necessary.*  *“No permits had to be obtained as the work was conducted by the USFS.”*

4. We note that you have stated that you will provide repository information for your data at acceptance. Should your manuscript be accepted for publication, we will hold it until you provide the relevant accession numbers or DOIs necessary to access your data. If you wish to make changes to your Data Availability statement, please describe these changes in your cover letter and we will update your Data Availability statement to reflect the information you provide.

***Response:*** *USGS requires all data used for scientific publications be released at the time of publication. The data supporting this manuscript will be made available at DOI:* [*https://doi.org/10.5066/P9XON1P4*](https://doi.org/10.5066/P9XON1P4) *once the dataset and manuscript have USGS approval for publication.*

5.  We note that Figures 1 and 2 in your submission contain [map/satellite] images which may be copyrighted. All PLOS content is published under the Creative Commons Attribution License (CC BY 4.0), which means that the manuscript, images, and Supporting Information files will be freely available online, and any third party is permitted to access, download, copy, distribute, and use these materials in any way, even commercially, with proper attribution. For these reasons, we cannot publish previously copyrighted maps or satellite images created using proprietary data, such as Google software (Google Maps, Street View, and Earth). For more information, see our copyright guidelines: <http://journals.plos.org/plosone/s/licenses-and-copyright>.

We require you to either (1) present written permission from the copyright holder to publish these figures specifically under the CC BY 4.0 license, or (2) remove the figures from your submission:

***Response****: The maps in figure 1 and 2 were created by the authors. QGIS and FragStats are both open source software, their logos are available for use.*

Reviewers' comments:

Review Comments to the Author

Reviewer #1: This well-written manuscript describes the recent invasion of Yersinia pestis (the causative agent of sylvatic plague) into Conata Basin, South Dakota. The paper summarizes data on prairie dog colony characteristics (e.g., size, shape, aggregation) before and after plague’s invasion. The paper is unique in some respects; prior studies have concentrated (mostly) on areas with historic, endemic plague, and have not included before-after comparisons (but see Eads et al. 2018 for an example from Conata Basin). On the other hand, the paper describes observable, documented phenomena that have been discussed for decades (plague-induced prairie dog population destruction). The manuscript is fascinating and builds upon additional observations/studies that might be discussed in a manner to reduce the tone of novelty.

***Response:*** *Thank you. We have toned down the claims of novelty (not required by PLoS One for publication) throughout.*

General comment: The paper concentrates on colony size, shape, and aggregation, which is understandable and appropriate; the underlying data are well suited from such an assessment. That said, additional colony characteristics are important. For example, black-footed ferrets selectively use areas with high densities of active burrow openings (refuge) and prairie dog prey (Biggins et al. 2006, Eads et al. 2011, Livieri and Anderson 2012). These characteristics are extremely important for ferrets, and likely other species. Eads et al. (2018) provide some information on fine-scale changes in burrow/prey density before and after plague’s invasion of Conata Basin. Additional information may be available from those authors and, more generally, groups involved in long-term prairie dog and ferret conservation in the study area.

***Response:*** *We are aware of other efforts to understand prairie dog colony dynamics in the area. However, none of those efforts had the same spatial or temporal scale of this analysis and would require substantial subsetting of included colonies. We have included a statement in the discussion about other metrics that may be important for black-footed ferret recovery (besides size, shape, and aggregation).*

General comment: The paper concentrates on epizootic plague. Enzootic plague (i.e., transmission between epizootics) is of extreme importance in black-footed ferret and prairie dog conservation (Biggins et al. 2010, Matchett et al. 2010). The authors might briefly mention and describe enzootic plague in the Discussion and emphasize the importance of enzootic plague for wildlife conservation.

***Response:*** *We have added a sentence about enzootic plague in the discussion. Because we only evaluated 7 years post epidemic, and this was a major fragmenting event (naive populations with invading disease), we felt interpreting the reduced recovery of prairie dog colonies after the outbreak in light of enzootic plague was premature. However, if the time series of colony sizes continues to be collected, it may be possible to evaluate whether there is recovery in between epizootics and thus find evidence for or against the occurrence of enzootic plague in this region.*

General comment: The paper does not mention the decline of black-footed ferrets at Conata Basin. The paper might benefit from some information on the ferret decline (e.g., in the Discussion) given the site is managed (mostly) for ferret conservation. USFS and Prairie Wildlife Research may provide useful information.

***Response****: We have added some context about management for Black-footed Ferrets throughout.*

Title: Possibly change “pathogen” to “disease”; the title concentrates on sylvatic plague, not Yersinia pestis.

***Response:*** *Changed*

Keywords: Please change “sciuridae” to Sciuridae.

***Response:*** *Thank you for catching that!*

Line 38: The authors suggest emerging infectious diseases are “not traditionally thought of as a source of disturbance”. The material might be modified; many authors have discussed ecological disturbances and transformations caused by invasive plague (e.g., Antolin et al. 2002, Kotliar et al. 2006, Eads and Biggins 2015).

***Response:*** *In the case of plague, this has been often discussed. However when thinking about the overall field of ecology, disease is rarely included as a disturbance. We agree with the reviewer that this perception is changing and adjusted our wording.*

Line 60: Possibly note that Cynomys occur in Mexico and Canada, as well as the US.

***Response:*** *Southern Canada and northern Mexico were added.*

Line 93 and elsewhere, including figures: “Introduction” might be changed to “invasion” to emphasize how plague (probably) arrived at Conata Basin (probably via ecological invasion, though human-assisted introduction is possible).

***Response:*** *Thank you for the suggestion. Adjusted throughout the manuscript.*

General comment: In 2007, there was some evidence of sylvatic plague on a colony near Highway 44 (sudden disappearance of prairie dogs documented by USGS-FORT field researchers; plague was not confirmed).

***Response:*** *Thank you for sharing that information. We added a comment to the discussion, explaining that* Y. pestis *might have been present prior to detection.*

Line 115: The authors might mention weather as another influence on prairie dog populations and colonies.

***Response:*** *Done*

Line 124: Proactive deltamethrin dusting started in 2005.

***Response:*** *We confirmed that there was some limited dusting on two colonies within the Conata Basin Complex in 2005-2007 for a research project with Dean Biggins.*

Line 124: To my knowledge, 1 of the authors (RG) was intimately involved in plague mitigation and research at the study site. The remaining authors might have been involved peripherally, for a short amount of time. In addition to deltamethrin, several other forms of experimental plague mitigation (vaccines and insecticides) were under use/testing at the study site, especially after plague’s invasion, and those mitigation measures should be discussed, as they might influence results from this study. The authors might contact other researchers intimately involved in plague mitigation and research at Conata Basin, and request help, so the paper fully summarizes all forms of plague mitigation and potential influences on the results. For example, how might the following have affected the results: research on an injectable plague vaccine with prairie dogs and small rodents (USGS-FORT unpublished data); research on an edible vaccine with prairie dogs (Rocke et al. 2017); research on a variety of insecticides (Eads et al. 2019)? How might flea resistance to deltamethrin (Eads et al. 2019) have affected the results?

***Response:*** *We acknowledge that the use of experimental treatments to protect prairie dogs, small rodents, and ferrets occurred on small experimental plots that may have been included in our study. However, most of these experiments were conducted in the latter years (for example the sylvatic plague vaccine trials were 2013 - 2016), and on a very small portion of the surveyed colony complexes (the plague vaccine/placebo plots together were 80 ha spread across 2 colonies). The data used in our analysis covers 1993 - 2015 and includes a total colony area of 23,290 ha (though that changes by year). We have added a comment in the methods acknowledging that other experiments were probably on-going in some areas.*

Line 138: Colonies adjacent to Badlands National Park are cutoff at the park boundary, even though some of the colonies extend into the Park. This phenomenon affects colony size and shape. How might this phenomenon affect the results?

***Response:***  *Looking more closely at the 91 selected colony complexes, about 15 have artificially constricted boundaries. If colonies grow and constrict in a non-uniform way (e.g. across all edges of the boundary), then we may have underestimated changes in colony shape and complexity for these 15 colonies. However, given this is a smallish subset of the total number of colonies, and the overall strength of the detected patterns, we suspect these artificial boundaries are not overly biasing our results. We have added this detail to the methods.*

Line 162: Possibly add justification for the use of 25 m resolution.

***Response:*** *Added that it is the default setting in ArcGIS 10.*

Line 191: Did the authors consider non-linear forms of “years since outbreak”?

***Response:*** *We did not, as the linear models were good fits for the dataset.*

Figure 2: This figure zooms into section B, which includes many colonies treated with deltamethrin and other experimental plague mitigation tools. Consequently, section B concentrates on areas with data that underestimate the effect of plague. The authors might consider adding another figure (even as a supplement) that depicts colony areas in 2007 (on the left) and 2009 (on the right), with no mapping for other years. The change was visually dramatic.

***Response:*** *We have included the recommended figure in the supplement.*

Figure 2: Were Heck Table and areas further to the west included in this study?

***Response:*** *Yes, Heck Table was included. We have added more spatial information as a supplementary figure and in the methods.*

Figure 2: Were areas north of the Badlands wall included in this study? If so, the analyses might include a variable for “south” or “north” of the Badlands wall (given plague has most dramatically affected areas south of the wall). The analysis might benefit from a separate assessment of data from south of the wall (alone; this should help to better illustrate the devastating effects of plague, which had not really manifested north of the Badlands wall).

***Response:*** *Yes, sites north of the wall were included. On visual inspection of the yearly shapefiles, there appears to be just as much fragmentation and reduction in colony size North of the wall as South of it. In addition, the North sites were not routinely surveyed until 2006.*

Thank you for the opportunity to review this interesting manuscript. I hope my comments are useful to the authors.

***Response:*** *Thank you for your comments! They were exceptionally insightful.*

Reviewer #2: Review of PONE-D-20-01395

An invasive pathogen, sylvatic plague, increases fragmentation of black-tailed prairie dog (Cynomys ludovicianus) colonies

Summary: The authors use a prairie dog dataset from Conata Basin, South Dakota to demonstrate how an introduced disease (plague) affects the general landscape characteristics of the prairie dog colonies. The paper is well written and presents quality research that is unique (i.e., before-after effect of plague on prairie dog colonies). The Introduction does an excellent job of describing the background, need for the study, why it is important, and the general objectives. The Methods are well written but need some clarification and justification, primarily in the Spatial Analysis, for the reader to better understand the metrics and how they were measured. My specific comments are below and include censoring the 1993 and 2006 data sets.

***Response:*** *We have run the analysis with and without 1993 and 2006. There was no change in the significance of the results nor generally the coefficients when removing these years. In addition, removing those years reduced our sample size from 91 to 67. Thus, we have chosen to keep them in the main manuscript but include the reduced models in the supplemental R markdown file.*

Figure 1 is excellent in demonstrating the workflow, but could use a little bit more explanation in the text or caption.

***Response:*** *Additional explanation text has been added to the caption. In addition, we have clarified the spatial analysis methods used throughout.*

Figure 2 provides a nice visualization of some of the input data, but is not as vital as Figure 1.

Figure 3 provides a good summary of the results.

Table 1 is appropriate.

Table 2 is relatively dense with information, but the only suggestions I have for improvement would be removal of 1993 and 2006.

The Data Analysis is appropriate and well executed. Results are complete and can take a few reads before fully understood. Because the data are rasterized, the reader has to be mindful that the numbers reported are not exactly what was measured on the landscape, but rather a quantitatively accurate generalization. The Discussion and Conclusion appropriately highlight the important findings and place them in the appropriate management context. The authors do a good job of explaining limitations and suggest further research. References may need a few edits. Supplemental Material table is appropriate.

***Response:*** *Thank you for the thorough review of our manuscript.*

Specific comments:

Line 61: “inhabit only 2% of their historic range…”. Estimates of prairie dog decline vary, thus I suggest a slight change in wording to account for variable estimates, such as “inhabit approximately 2% of their historic range…” See the summary of historic range estimates in Miller et al. 2007 Prairie dogs: an ecological review and current biopolitics. Journal of Wildlife Management 71:2801-2810.

***Response:*** *Textual change made and reference added*.

Lines 88-89: “Lastly, warm winters and wet springs…” For an alternate view, see Eads and Biggins 2017 Paltry past precipitation: predisposing prairie dogs to plague? Journal of Wildlife Management 81:990-998.

***Response:*** *Added.*

Lines 104-107: “All of the prairie dog colonies surveyed in the study area…” I suggest acknowledging that some prairie dog colonies were contiguous with other federal (i.e., Badlands National Park) or private lands and thus some colony borders analyzed in this study were straight lines (i.e., borders) between land ownership and not necessarily a true characteristic of the colony. Some readers may be confused by some of the straight line borders in Figure 2 and thus suspect that some colonies may be larger or have a different shape than what was analyzed.

***Response:*** *Included and clarified.*

Lines 110-114: “Beginning in 2005, Boundary Management Zones (BMZs) were used…” I agree that the consistent BMZ management from 2005-2015 should not be a problem in interpreting fragmentation post-2008. However, prior to 2005 there were often a significant amount of prairie dog colony area in the BMZ area. Did the authors remove the BMZ (i.e., remove the ½ mile) from the colonies prior to 2005? Otherwise the estimates of class area and other metrics (Table 1) presented in Table 2 would be inflated for any colonies prior to 2005.

***Response:*** *Only ~5 colonies of the 91 selected crossed into private lands and would have potentially been part of the BMZs. Of these, visual inspection did not show any clear difference in the location of the boundaries by year pre or post 2005.*

Lines 125-127: “The use of dusting…” I’m glad the authors acknowledge dusting but it certainly complicates interpretation of the results because it is an effective tool in increasing prairie dog survival at certain levels of plague circulation (Biggins et al. 2010 Vector control improves survival of three species of prairie dogs (Cynomys) in areas considered enzootic for plague. Journal of Vector-Borne and Zoonotic Diseases 10:17-26). Thus the probability of colonies persisting and/or maintaining during plague is possible by dusting. Because dusting is very labor-intensive, it can be difficult to treat entire colonies, potentially creating artificial borders between dusted and non-dusted areas. For instance, in Figure 2, Box B, the large yellow colony in the upper right quadrant has a diagonal line across from SW to NE. That line is very close where dusting occurred (green) and likely influenced the size and shape of that particular colony. Additionally, not every area was continually dusted every year (due to budget) or timing between annual dust applications varied.

***Response:*** *The expected effect of dusting was explicitly added to this section.*

Line 131: “…transverse…” I would suggest using a different word as transverse means crossing, often in perpendicular direction. I suggest using “record” or “trace” in this instance as the mappers followed the outline of the colony.

***Response:*** *Changed to record.*

Line 133: “coordinates every second.” For the years 1999-2005 the GPS units recorded a coordinate every 5 seconds (I created the data dictionary and did much of the mapping). Based upon the shape of the colonies in the 1996 coverage, I suspect it was also 5 seconds. Griebel was present 2007-2015 and oversaw all the mapping and can confirm if those years were mapped using 1 or 5 seconds between locations. While this may seem trivial, the interval between locations and the speed traveled can affect the overall shape of a polygon.

***Response:*** *Changed to 1 to 5 seconds. Also, thank you for all your hard work, we feel privileged to work with the dataset!*

Lines 135-137: “This method was used to estimate colony size…” The 1993 coverage was not estimated by GPS ground mapping, but through aerial photography (see Schenbeck and Myhre 1986 Aerial photography for assessment of black-tailed prairie dog management on the Buffalo Gap National Grassland, South Dakota. US Forest Service Technical Report 86-7). I would suggest not using the 1993 coverage because it was derived from aerial photography, the area was open to prairie dog shooting (closure started in 1998, with the exception of a few small areas), and many areas were subject to poisoning, which certainly can impact prairie dog populations. Along those same lines, I would suggest eliminating use of the 2006 coverage as well because it was derived using a different method (aerial photography).

***Response:***  *Clarified our recreational shooting statement in line 108-110. Also added, 1993 and Schenbeck reference to the text. We conducted the analysis without 1993 and 2006, please see our response to above comment about why we decided to leave these in the analysis.*

Line 145: “between the years 1993 and 2015”. Again, I would suggest not using the 1993 and 2006 coverage layers because they were collected under a different methodology. Looking at Table 2, there is a marked decrease in some of the metrics between 1993 (aerial photography) and 1996 (GPS ground) that could be an artifact of poisoning, shooting, or methodology. Similarly, the 2006 metrics do not fit the general known pattern (prairie dog colonies increased in size from 1999 to 2007). From my experience on the ground, the colonies did not decrease in size/distribution from 2006-2007 (as Table 2 would suggest and the authors state later in the paper on Line 211).

***Response:*** *We conducted the analysis without 1993 and 2006 (included in the supplementary R code), see response above.*

Lines 145-150: “The colonies in the composite map were buffered…” I understand and support the concept of using a composite map to facilitate the consistent tracking of prairie dog colonies that coalesce/fragment and I agree that 69m is a reasonable daily distance for a prairie dog to move WITHIN a prairie dog colony, but not out from a prairie dog colony. In the Conata Basin, colonies are often separated by barriers that inhibit the daily movements of prairie dogs. The three darkest colonies in Figure 2, Box B are less than 138m from each other, but are separated by deep drainages that are only crossed by prairie dogs during dispersal (certainly not on a daily basis). It’s unclear as to why a buffer is needed from the edge of the colony and some further description/explanation would be appreciated. I’m also having difficulty understanding how the authors had 425 colonies in the composite map. In 2007, there were 40 individual colonies in Box A of Figure 2 (i.e., 40 polygons), which then buffered by 69m results in 9 colonies (i.e., polygons). How did the authors derive 425 individual colonies from a composite map, particularly after buffering by 69m? I may be misunderstanding the methodology.

***Response:*** *We added language to clarify that natural barriers were not accounted for and colonies might have been counted as one even when split by natural barriers, like the deep drainages. The buffering was only used to identify colony complexes through time of the original mapped colony boundaries by year. 425 were the total number of unique colony complexes before our selection criteria were applied. Once we selected colonies with at least 3 surveys, with one before and one after plague arrival we only had 91 unique colony complexes. We have tried to clarify this part of the methods throughout.*

Lines 155-156: “Selected colonies were sampled…” Please clarify if the sampling was random, stratified, systematic, etc. If I understand this correctly, for each colony, a value was chosen from one coverage year prior to plague and two coverage years after plague. These values were used in the models (later described). Thus the data for colony #28 could be from 2002, 2009, and 2015. And then the data for colony #29 could be from 2005, 2011, and 2013. Why not use all of the data?

***Response:*** *We use all the years observed for colonies with three or more observations.  We clarified this in our methods section. No subsampling was conducted.*

Line 157: “…after plagues arrival…” Suggest a change to “…after the arrival of plague…”

***Response:*** *Language was adjusted.*

Lines 161-162: “…develop raster-based GIS files with a resolution of 25m…” Is this the cell size and then each patch is a contiguous set of cells? I suggest a simple description to help the reader better understand the definition of a patch.

***Response:*** *25 m is the default cell size for conversion of shapefiles to rasters in ArcGIS 10. It is possible to do smaller cell sizes, but it quickly needs more computational power and creates extremely large file sizes. Given the overall scale of the landscape, we felt 25 m was appropriate detail for the boundaries.*

Line 168: I suggest providing a web citation for FRAGSTATS. McGarigal, K., SA Cushman, and E Ene. 2012. FRAGSTATS v4: Spatial Pattern Analysis Program for Categorical and Continuous Maps. Computer software program produced by the authors at the University of Massachusetts, Amherst. Available at the following web site: <http://www.umass.edu/landeco/research/fragstats/fragstats.html>

***Response:*** *Done.*

Line 204: “…425 unique colonies were identified…” Please clarify how this number was determined per comments above.

***Response:*** *Done.*

Line 209: “The mean number of patches per colony decreased…” The mean number of patches per colony appeared to be stable for 1996, 1999, and 2002. Then a significant increase occurred in 2004, followed by a significant decrease in 2005. Undoubtedly, the colonies were expanding during this time frame and coalescing, but the 2004 data presented in Table 2 appears to be an anomaly? Also, the mean number of patches during the plague years (2009, 2011, 2013, 2015) does increase over time, but not to the same degree as 2002 to 2004.

***Response:*** *One of the columns of our table was cut-off in the reviewed manuscript. This included the number of colony complexes included by year.*

Line 211: As noted above, I would suggest reconsidering the use of the 2006 data because of the methodology that could account for significant differences. If the 2006 data is used, then it suggests that the total area of prairie dogs decreased in 2007, before the known invasion of plague.

***Response:*** *We re-ran the analysis without 1993 and 2006, showing no difference in the interpretation of the results. By excluding these years, we reduce our overall sample size, so we have selected to keep them included in the main MS. We have added this analysis to the supplement.*

Line 216: Comments on Table 2. In the caption for Table 2, I would suggest adding in a description that this table contains data for the 91 selected colonies, not all of the colonies in the study area. It would be helpful to also describe the data as rasterized so that readers don’t interpret the numbers as actual vector-mapped colony sizes. The definitions of “colony” and “patch” should be clarified for the Table. As I read it, the No. of colonies is the number of composite colonies with a unique ID (out of a total of 425?). In the flowchart of Figure 1, which illustrates the workflow well, the last component visually suggests that patch is a contiguous set of 25m grid cells. If that is the case, then any contiguous prairie dog colony (vector or raster) on the landscape comprises one patch? The Table reports 500 patches in 2015 but the 2015 GIS coverage that I have only has 400 total prairie dog colonies (polygons). Can a contiguous polygon be split in the vector-to-raster process?

***Response:*** *We have updated the table caption to explain that this is the summarized metrics from FRAGSTATS of the by year rasters. Yes, it is possible for a colony to be split into multiple patches if it has a long-skinny section that is not at least half of a cell size (so less than 12.5 m squared). This conversion effect should not influence the results as it is the same for all years.*

Line 298: “…limitation…” Another suggestion could be cascading effects from plague that were difficult to measure, such as increased rainfall (see Figure 11 in Kempema et al. 2015 Colony acreage and distribution of the black-tailed prairie dog in South Dakota, 2012). Drought conditions from 1999-2007 facilitated growth of colonies (e.g., prairie dogs had to seek more forage over a greater area). Above-average rainfall in 2008 (the same year plague invaded) likely also stimulated vegetation growth and the prairie dogs that survived plague were further limited by increased vegetation, thus furthering declines in area, shape, etc. Undoubtedly, plague caused a significant reduction in colony area, shape, and contiguity, but we often speculated that increased rainfall could exacerbate the effects of plague through vegetative growth.

***Response:*** *We have added this as a potential confounding factor in our discussion of fragmentation post-plague.*

Lines 314-315: “If we had been able to determine…” From my understanding, every colony was revisited during each mapping effort for 2002-2015, thus any colonies that did not appear after 2008 were truly extirpated.

***Response:*** *This is not correct. There was mapping in 1993, 1996, 1999, 2002, 2004 and 2005 which only included the Conata Basin area except for the 2006 aerial survey, which included the entire District. Efforts after 2007 included the entire District each year that was mapped. Survey efforts excluded the eastern and northern portions of the study area until 2007.  We felt it was important that we have at least 3 time points per colony because PD colonies are very dynamic through time (even in the absence of plague), and we wanted to make sure we captured some of this variation.*

Line 322: “…consisted of one very large colony…” This is confusing related to the 425 colonies reported earlier in the manuscript.

***Response:*** *We have clarified in text. Of the 91 colony complexes included in the analysis, one is much larger and had the largest number of individual patches compared to all the others. Thus, we wanted to ensure that our results were not skewed by this one large outlier.*

Lines 324-328: “In addition, colony area…” The authors support reasoning for excluding the 2006 coverage from the data set altogether (and the 1993 data set too). I would suggest that keeping the 1993 and 2006 data sets unnecessarily complicates the manuscript and the removal would strengthen the manuscript.

***Response:*** *See above response.*

Line 341: “…appearance…” The authors consistently used the word “introduction” in reference to plague and I would suggest using the same word here.

***Response:*** *Per suggestion of another reviewer, this word was changed to invasion.*

General comments: The GIS coverage data sets for 2007, 2009, 2011, 2013, and 2015 all excluded water sources on the prairie dog colonies (i.e., stock dams) but the prior data sets did not exclude the water sources. On the more recent data sets, this would look like holes within the prairie dog colony, whereas the prior data sets do not have any such holes, although the stock dams were present. It may be helpful to eliminate those stock dams from the prior years, otherwise those would be rasterized cells that would be considered prairie dog colonies during the 1993-2006 period and then would not be colonies during the 2007-2015 period. That could introduce a small level of bias in the data set.

***Response:*** *We visually inspected the datasets across years, and could not identify any instance of a colony with and without water sources. Perhaps colonies with water sources were not included in our selected 91 complexes? This, added to the 25 m raster conversion, makes this unlikely to affect our results.*

The References may require some revisions (all Caps in some citations [2], capital letters in some article names [3,4,13,18,21,22,25,32,37,38,39]), although I am not entirely familiar with PLOSOne formatting requirements.

***Response:*** *Done.*
