# Supplementary material for: An invasive disease, sylvatic plague, increases fragmentation of black-tailed prairie dog (Cynomys ludovicianus) colonies
Source: PLoS One. 2020 Jul 23;15(7):e0235907. doi: 10.1371/journal.pone.0235907 (PMC7377483; doi:10.1371/journal.pone.0235907)
Supplement: Attachment — Submitted filename: Response to Reviewers2.docx [file pone.0235907.s007.docx]

Dear Dr. Becker,

Thank you. We addressed comments by the reviewers and additional comments provided by the USGS review process. Please find below our response to the reviewer’s comments, a track changes document and the document with all changes accepted.

Your Sincerely,

Kathrine L.D. Richgels

Reviewers' comments:

Reviewer's Responses to Questions

**Comments to the Author**

1. If the authors have adequately addressed your comments raised in a previous round of review and you feel that this manuscript is now acceptable for publication, you may indicate that here to bypass the “Comments to the Author” section, enter your conflict of interest statement in the “Confidential to Editor” section, and submit your "Accept" recommendation.

Reviewer #1: (No Response)

Reviewer #2: All comments have been addressed

2. Is the manuscript technically sound, and do the data support the conclusions?

The manuscript must describe a technically sound piece of scientific research with data that supports the conclusions. Experiments must have been conducted rigorously, with appropriate controls, replication, and sample sizes. The conclusions must be drawn appropriately based on the data presented.

Reviewer #1: Partly

Reviewer #2: Yes

3. Has the statistical analysis been performed appropriately and rigorously?

Reviewer #1: Yes

Reviewer #2: Yes

4. Have the authors made all data underlying the findings in their manuscript fully available?

The [PLOS Data policy](http://www.plosone.org/static/policies.action#sharing) requires authors to make all data underlying the findings described in their manuscript fully available without restriction, with rare exception (please refer to the Data Availability Statement in the manuscript PDF file). The data should be provided as part of the manuscript or its supporting information, or deposited to a public repository. For example, in addition to summary statistics, the data points behind means, medians and variance measures should be available. If there are restrictions on publicly sharing data—e.g. participant privacy or use of data from a third party—those must be specified.

Reviewer #1: Yes

Reviewer #2: Yes

5. Is the manuscript presented in an intelligible fashion and written in standard English?

PLOS ONE does not copyedit accepted manuscripts, so the language in submitted articles must be clear, correct, and unambiguous. Any typographical or grammatical errors should be corrected at revision, so please note any specific errors here.

Reviewer #1: Yes

Reviewer #2: Yes

6. Review Comments to the Author

Please use the space provided to explain your answers to the questions above. You may also include additional comments for the author, including concerns about dual publication, research ethics, or publication ethics. (Please upload your review as an attachment if it exceeds 20,000 characters)

**Reviewer #1:** This interesting manuscript builds upon existing observations/studies, demonstrating strong influences of plague on prairie dogs. Thank you for the opportunity to review the revision. The authors addressed many of my original comments. Thanks to the authors for their efforts. I hope the following comments are helpful.

Funding and Ethics statements: Operational plague mitigation at Conata Basin has been, and continues to be, highly collaborative. A variety of groups have provided support . It seems the collaborators and funders should be acknowledged (USFS, Prairie Wildlife Research, USFWS, USGS-FORT, World Wildlife Fund, etc.).

**Reply:** *Thank you. Author R. Griebel was at USFS Conata Basin from 2007 – 2015, he was unaware of the contribution of these groups before or after his time there. We have added this to the acknowledgments.*

Line 65: Although this paper concentrates on a plague epizootic at the Conata Basin, it seems important to emphasize that “enzootic plague” (i.e., plague transmission and host mortality at sub-epizootic levels) also affects prairie dogs and associated species. In fact, enzootic plague may be the primary threat to endangered black-footed ferrets – epizootics are infrequent, and enzootic plague reduces ferret survival by at least 200% during inter-epizootic periods (Matchett et al. 2010). The introduction and discussion should emphasize the importance of enzootic plague so the reader understands the importance of this prominent, albeit less understood portion of plague cycles.

**Reply:** *The presence of Y. pestis was added to the paragraph as a post-plague die-off problem. While we agree with the reviewer that enzootic plague is important for black-footed ferret survival and long-term species recovery, the manuscript is focused on an epizootic event affecting prairie dogs. We feel that a mention of enzootic plague is appropriate (as updated in the introductory text), but that an extensive review of the topic and its implications would not be appropriate for the context of this particular study.*

Lines 74-75: The authors suggest, “the arrival of Y. pestis on a particular prairie dog colony generally leads to complete colony collapse”. This statement depends on the definition of “colony collapse”. Generally speaking, for many prairie dog colonies, and large colonies in particular, some prairie dogs remain after an initial epizootic. That said, declines may be defined as “collapse” from the perspective of ferret conservation (if only a few prairie dogs remain, the ferrets will die). The authors cite Pauli et al. (2006) as support. If this sort of statement remains in the paper, the authors should also cite Tripp et al. (2017), who detected functional colony collapse in the presence of epizootics.

**Reply:** *The wording was rephrased to population declines.*

Line 76: The authors suggest some colonies are “unaffected”. However, low-level enzootic plague can cause population perturbations that are imperceptible to humans, i.e. unless experiments are conducted (Biggins et al. 2010, Matchett et al. 2010). Seemingly unaffected colonies are probably affected at some level over time, even when epizootics are not observed on those colonies. Enzootic plague deserves greater attention in this manuscript.

**Reply:** *Changed wording to remove implication that some colonies are unaffected.*

Line 77: Please change to “re-emerging in epizootic form”.

**Reply:** *Changed to “plague epizootics are cyclical…”*

Line 81: Please change to “low levels of endemicity that are difficult to detect but, nonetheless, ecologically important” or something similar to emphasize the importance of enzootic plague.

**Reply:** *Rephrased and added reference to Matchett et al. 2010*

Lines 107 and 389: The authors suggest the work was completed by USFS. However, the number of collaborative groups and agencies is very long (even when considering dusting alone). For example, USFWS provided A LOT of funding for dusting. NPS and USFS work in close collaboration on plague mitigation. Prairie Wildlife Research, USGS-FORT, and World Wildlife Fund contributed to the overall team effort.

**Reply:** *Line 107 - we say the land is managed by the USFS, which is correct. However, we have updated the acknowledgement to recognize the consortium of partners who supported black-footed ferret recovery and plague management at Conata Basin.*

Line 115: The authors suggest the BMZ was consistently managed. In some (many) cases, areas within the BMZ were not consistently managed – they were poisoned etc. only when the areas extended to within ½ mile of private lands and (usually) only when private landowners complained.

**Reply:** *Changed to better reflect that we don’t think BMZs affected our results because they affected a small number of the total selected colonies (5/91) and there was no signal of this management in the shapefiles.*

Line 118: Ferrets were first reintroduced to the area in 1994, and 146 captive-born kits were reintroduced into Conata Basin during 1996-1999 (Livieri 2006, Livieri and Anderson 2012).

**Reply:** *We added the additional ferret reintroduction events and adjusted the citation.*

Line 122: Please remove “)” at the end of the sentence.

**Reply:** *Removed*

Line 124: Tularemia should NOT be mentioned as a source of large-scale impacts, because tularemia is only known to affect pockets of prairie dogs, not larger colonies (Cherry et al. 2019).

**Reply:** *Tularemia was removed.*

Lines 130-131: Eads et al. (2019) tested a variety of pulicides on multiple colonies, including colonies in the core of Conata Basin, for multiple years.

**Reply:** *Our study period included 1993 - 2015, the study cited by the reviewer started in summer/fall of 2015. Thus, depending on the timing of the colony surveys, Eads et al. 2019 had no or minimal temporal overlap to the data presented here.*

Lines 141-143: Biologists confirmed, via ground truthing, that some of the aerial-mapped colonies were inactive and therefore should not be included in the current study (because those “colonies” were not really prairie dog colonies – only old burrow mounds remained due to poisoning and other factors).

**Reply:** *In response to reviewer 2’s comments during the first round of review, we ran the analysis with and without 1993 and 2006. There was no change in the significance of the results nor generally the coefficients when removing these years. In addition, removing those years reduced our sample size from 91 to 67. Thus, we have chosen to keep them in the main manuscript but include the reduced models in the supplemental R markdown file.*

Lines 151-156: The authors suggest black-tailed prairie dogs use an average area/distance of 69 m per day. They cite data from Rocke et al. (2017). That particular dataset is very large and includes data that were kindly provided by a variety of state, government, non-profit, and Tribal groups. Most of those data are not suited for an evaluation of daily movements, and insufficient for an evaluation of territory sizes (because trapping was of short duration at most sites, within and between years). It’s important to note, data from Rocke et al. (2017) were collected at multiple sites affected by epizootic plague, and plague outbreaks “break” the prairie dog social structure and favor longer movements by animals (Biggins and Eads 2019). When considering more robust datasets, black-tailed prairie dogs move over much shorter distances (e.g., 20-40 m or so, often less than 15 m over monthly periods).

**Reply:** *The buffering was used to identify colony complexes through time from the shapefiles of mapped colony boundaries by year. Some amount of buffering was necessary to link colonies that fragmented or shifted spatially through time. Published information on prairie dog home ranges was actually quite difficult to find at the time of the analysis (we searched the literature for manuscripts that evaluated home range size and were unsuccessful, leading us to use the data we reference above). However, since we began this project a paper supporting the distance used (from a study that estimated coterie sizes of prairie dog complexes in South and North Dakota) was published. We have added it as support for the distance we chose.*

Line 164: The authors commonly use “colony complexes” when referring to single colonies (I think). Complexes were defined by Biggins et al. (1993) and represent collections of colonies in close proximity. If the authors are referring to collections, they might cite Biggins et al. (1993) or provide an alternative definition for their purposes. If the authors are referring to single colonies, then “complexes” should be removed from the manuscript (except when referring to the larger Conata Basin complex).

**Reply:** *The authors understand the confusion and we had similar concerns. However to address concerns by reviewer 2 on how to interpret the represented metrics, we chose to use colony = patch, and colony complex = class. We specified this in the methods: “We then used a spatial join to transfer the unique ID of the spatially distinct merged colonies to the yearly shapefiles and refer to them as colony complexes (as depending on the year, there may be one or more spatially distinct colonies with the same unique ID), thus ensuring that patches, referred to as colonies, within each unique colony complex could be tracked through time.”*

Line 218: Please explain the cutoff of 2 AIC units. What happens if the authors use the more conservative AICc for small sample sizes?

**Reply:** The *2 AIC units follow the guidelines of Burnham and Anderson 2002 for model selection and multi-model inference. Since 567 observations were used for the reported analyses and we only include one parameter in our model the difference between AICc and AIC are a matter of decimals.*

Line 236 and Results in general: In the discussion, the authors might emphasize that rasterized colony boundaries will undoubtedly overestimate colony sizes (i.e. prairie dogs occupied less acreage than suggested by indices in this paper). This is an important point, because increasing prairie dog acreage may lead to increased complaints from private landowners. USFS and collaborators have reported on colony acreage elsewhere – someone might use this paper to refute previous reports (which would be inappropriate and unproductive).

**Reply:** *That is indeed something we would like to avoid. Since we selected for certain colonies, we do not provide an estimate for the full Conata Basin complex and we hope it will not be interpreted this way.* *In addition, rasterization can both over and underestimate colony size, as a pixel is considered part of the colony if >1/2 of the pixel area is occupied by the shapefile (so depending on colony shape and how it crosses the pixel grid, it can be either over or underestimated). We added a sentence to the discussion clarifying that the numbers reported in this study should not be used as an estimate of colony occupancy across the landscape.*

Line 294: The authors suggest the local ferret population declined by 1/3. The population declined from >300 down to about 35 (when considering the entire epizootic process).

**Reply:** *We adjusted the estimate to a reduction of 75% as described in the black-footed ferret recovery plan.*

Line 372: The authors suggest colony maps might be used to evaluate the efficacy of plague mitigation tools. Regarding the current study, it is important to note, after the epizootic most of the remaining colonies had been dusted with 0.05% deltamethrin dust (starting in 2005, in some cases, and continuing through this study). This study shows that 0.05% deltamethrin is a highly effective plague mitigation tool – currently, deltamethrin dust is the best tool (by far) for ferret conservation. The authors should highlight this important point in their manuscript, given their results are highly influenced by dusting (in the absence of dusting, few to no colonies would have remained in the “post-epizootic” period).

**Reply:** *We know that 4,000-6,000 ha were treated annually after 2008. Unfortunately we are missing two pieces of data that would allow us to evaluate this effect, (1) we do not have a complete record of which colonies were treated and when, and (2) the colonies were not surveyed consistently before 2007, and so it was not possible to estimate the extirpation of colonies due to plague (as opposed to other natural instances, had the time series been more robust). Though we agree with the reviewer that dusting most likely contributed to persistence of prairie dogs on the landscape in Conata Basin, and have made this statement stronger in the discussion.*

Lines 385-387: The authors could cite McDonald et al. (2011) [USGS Scientific Investigations Report 2011-5063]. The supplement suggests research is needed on managed and non-managed habitat to assess plague effects on a variety of species/systems.

**Reply:** [*https://pubs.usgs.gov/sir/2011/5063/*](https://pubs.usgs.gov/sir/2011/5063/) *Added.*

**Reviewer #2**: Thank you for addressing my concerns with the manuscript. The Methods, Spatial Analysis, and additional figures provide a more clear understanding. A few additional minor changes are suggested below:

Line 122: There appears to be an extra ")" after "[34]"

***Reply:*** *Removed*

Lines 125-125: As noted by the other reviewer, dusting began in 2005 on 7,000 acres (8 colonies), and repeated in 2006 and 2007 on 1,000 acres (2 colonies). None of the dusting was directly associated with Dean Biggins’ project, but was prophylactic protection and testing equipment/methods for the possible invasion of plague.

***Reply:*** *Thank you for informing us!* *R. Griebel did not begin working at USFS Conata Basin until 2007, and was unaware of these efforts. However, he found documentation of this effort and we have added these details to the methods.*

Line 346-347: “it is possible that it was circulating well before the first confirmed case.” I would suggest defining what “well before” means. We monitored prairie dog colonies for apparent signs of plague (e.g., burrow activity, colony activity), tested prairie dog carcasses, and measured carnivore seroprevalence. One seropositive coyote was detected in 2005 but no changes in prairie dog activity were documented. Three dead prairie dogs were tested in fall 2007 that were negative, but in the area that plague was confirmed in May 2008. Also, a few fleas collected in fall 2007 contained plague DNA. Thus I would estimate that it was no longer than 1 year before the first confirmed case. I would suggest changing the sentence to read "it is possible that it was circulating up to 1 year before the first confirmed case."

***Reply:*** *Remove “well” from the sentence.*

Line 348-349: “Because this dataset was collected during routine management for black-footed ferrets and prairie dog recovery plans”. I suggest changing the wording to “Because this dataset was collected during management of prairie dog colonies for black-footed ferret recovery…” There is not a recovery plan for prairie dogs as they are not a listed species.

***Reply:*** *Reword as suggested.*

7. PLOS authors have the option to publish the peer review history of their article ([what does this mean?](https://journals.plos.org/plosone/s/editorial-and-peer-review-process#loc-peer-review-history)). If published, this will include your full peer review and any attached files.

If you choose “no”, your identity will remain anonymous but your review may still be made public.

**Do you want your identity to be public for this peer review?** For information about this choice, including consent withdrawal, please see our [Privacy Policy](https://www.plos.org/privacy-policy).

Reviewer #1: No

Reviewer #2: Yes: Travis Livieri

[NOTE: If reviewer comments were submitted as an attachment file, they will be attached to this email and accessible via the submission site. Please log into your account, locate the manuscript record, and check for the action link "View Attachments". If this link does not appear, there are no attachment files to be viewed.]

While revising your submission, please upload your figure files to the Preflight Analysis and Conversion Engine (PACE) digital diagnostic tool, <https://pacev2.apexcovantage.com/>. PACE helps ensure that figures meet PLOS requirements. To use PACE, you must first register as a user. Registration is free. Then, login and navigate to the UPLOAD tab, where you will find detailed instructions on how to use the tool. If you encounter any issues or have any questions when using PACE, please email us at figures@plos.org. Please note that Supporting Information files do not need this step.
